# Effect of Physical Activity, Social Support, and Skills Training on Late-Life Emotional Health: A Systematic Literature Review and Implications for Public Health Research

**DOI:** 10.3389/fpubh.2014.00213

**Published:** 2015-04-27

**Authors:** Mark B. Snowden, Lesley E. Steinman, Whitney L. Carlson, Kara N. Mochan, Ana F. Abraido-Lanza, Lucinda L. Bryant, Michael Duffy, Bob G. Knight, Dilip V. Jeste, Katherine H. Leith, Eric J. Lenze, Rebecca G. Logsdon, William A. Satariano, Damita J. Zweiback, Lynda A. Anderson

**Affiliations:** ^1^Department of Psychiatry and Behavioral Sciences, University of Washington School of Medicine, Seattle, WA, USA; ^2^Health Promotion Research Center, University of Washington, Seattle, WA, USA; ^3^University of Washington School of Nursing with Environmental Health Focus, Seattle, WA, USA; ^4^Adolescent Medicine, Seattle Children’s, Seattle, WA, USA; ^5^Department of Sociomedical Sciences, Columbia University Mailman School of Public Health, New York, NY, USA; ^6^Department of Community and Behavioral Health, Colorado School of Public Health, University of Colorado Denver, Aurora, CO, USA; ^7^Department of Educational Psychology, Counseling Psychology Program, Texas A&M University, College Station, TX, USA; ^8^Davis School of Gerontology and Department of Psychology, University of Southern California, Los Angeles, CA, USA; ^9^Sam and Rose Stein Institute for Research on Aging and Department of Psychiatry, University of California San Diego, San Diego, CA, USA; ^10^College of Social Work, University of South Carolina, Columbia, SC, USA; ^11^Department of Psychiatry, Washington University School of Medicine, St Louis, MO, USA; ^12^Department of Psychosocial and Community Health, University of Washington School of Nursing, Seattle, WA, USA; ^13^School of Public Health, University of California Berkeley, Berkeley, CA, USA; ^14^Division of Chronic Disease and Injury Prevention, Michigan Department of Community Health, Lansing, MI, USA; ^15^Healthy Aging Council and Health Equity Council, National Association of Chronic Disease Directors, Atlanta, GA, USA; ^16^National Center for Chronic Disease Prevention and Health Promotion, Centers for Disease Control and Prevention, Atlanta, GA, USA; ^17^Rollins School of Public Health, Emory University, Atlanta, GA, USA

**Keywords:** mental health, aged, health promotion, review

## Abstract

**Purpose:**

Given that emotional health is a critical component of healthy aging, we undertook a systematic literature review to assess whether current interventions can positively affect older adults’ emotional health.

**Methods:**

A national panel of health services and mental health researchers guided the review. Eligibility criteria included community-dwelling older adult (aged ≥ 50 years) samples, reproducible interventions, and emotional health outcomes, which included multiple domains and both positive (well-being) and illness-related (anxiety) dimensions. This review focused on three types of interventions – physical activity, social support, and skills training – given their public health significance and large number of studies identified. Panel members evaluated the strength of evidence (quality and effectiveness).

**Results:**

In all, 292 articles met inclusion criteria. These included 83 exercise/physical activity, 25 social support, and 40 skills training interventions. For evidence rating, these 148 interventions were categorized into 64 pairings by intervention type and emotional health outcome, e.g., strength training targeting loneliness or social support to address mood. 83% of these pairings were rated at least fair quality. Expert panelists found sufficient evidence of effectiveness only for skills training interventions with health outcomes of decreasing anxiety and improving quality of life and self-efficacy. Due to limitations in reviewed studies, many intervention–outcome pairings yielded insufficient evidence.

**Conclusion:**

Skills training interventions improved several aspects of emotional health in community-dwelling older adults, while the effects for other outcomes and interventions lacked clear evidence. We discuss the implications and challenges in moving forward in this important area.

## Introduction

Emotional health is increasingly viewed as a multidimensional construct that includes both positive and illness-related dimensions. Hendrie et al. (1) characterized emotional health as self-efficacy, depression, hostility and anger, anxiety, psychological stress, optimism, self-esteem, quality of life, and other domains assessed by multidimensional measures. A report (2) using data from the Behavioral Risk Factor Surveillance System (BRFSS) (3) identified six indicators reflecting positive and illness-related emotional health outcomes in older adults: social and emotional support; life satisfaction; frequent mental distress; current depression; lifetime diagnosis of depression; and lifetime diagnosis of anxiety disorders.

Mental health is increasingly viewed as part of public health’s mission, as important as physical health in contributing to overall health and well being (2). Epidemiologic data links a range of health outcomes, particularly mortality and cardiovascular disease, to emotions (1). Despite the public health importance, little is currently known about the effectiveness of interventions to promote emotional health in community-dwelling older adults. One of the few available reports (4) reviews studies from UK, finding some evidence to support significant small-to-moderate improvements in emotional health from select exercise programs including mixed exercise programs, strength and resistance, aerobic, walking, and individually targeted health promotion interventions. However, it also indicated a clear shortage of robust evidence for effective programs to improve late-life emotional health.

Although this review (4) addressed several important questions, a more rigorous review of the scientific literature is warranted. The primary objective of this systematic literature review was to identify interventions to promote emotional health of older adults aged 50 years and older. We sought to expand Windle and colleagues work by encompassing a wider range of community-based interventions, including more than UK-based studies, examining multiple domains of emotional health incorporating both positive and illness-related dimensions, and addressing community-dwelling older adults.

## Materials and Methods

### Data sources

#### Conceptual framework and definition of emotional health

**Figure 1:**
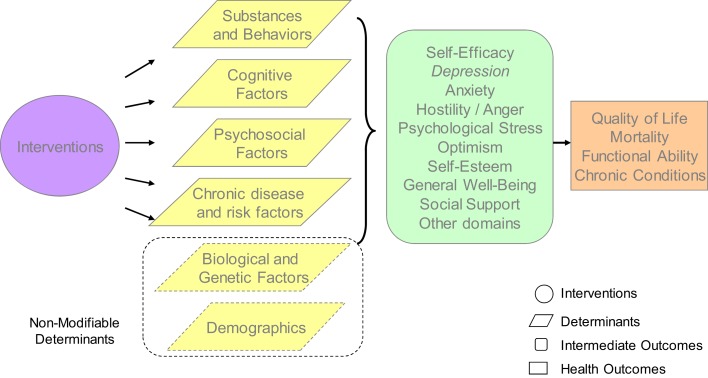
**Conceptual framework**.

This review used the NIH’s Cognitive and Emotional Health Project (1, 5) to guide the development of our conceptual framework and definition of emotional health (Figure 1). Interventions to promote emotional health can influence various determinants of emotional health. These determinants include substance use and other behaviors, cognitive factors, psychosocial factors, emotional factors, and chronic conditions. Risk and protective factors for emotional health also included less modifiable biological and genetic factors and demographics. For the purpose of this review, we focused on interventions aimed at modifiable determinants.

Borrowing from Hendrie and colleagues, we defined emotional health comprehensively as including both emotion regulation concepts (e.g., the ability to control/regulate emotions) and emotion intelligence (e.g., the ability to recognize and use emotions constructively). Most importantly, emotional health is multidimensional, involving positive mental health constructs, such as life satisfaction as well as illness-related domains such as anxiety. We used Hendrie and colleagues’ emotional health domains (1) and added “general well being” and “social support,” given research describing the relevance of these constructs to emotional health (6–8). The emotional health constructs used in this review are provided in the first row of Table 1. Finally, based on the literature, the conceptual model included longer term health outcomes associated with emotional health, including reductions in mortality and improvements in functional ability, morbidity of chronic conditions, and overall quality of life (entailing both physical and emotional well being).

**Table 1:** **Search terms used in electronic searches**.

Construct	Search terms	
Emotional health	Emotional health	*Interpersonal trust*
	Self-efficacy	*Positive Energy*
	Locus of control	Happiness
	Personal control	*Contentment*
	Personal mastery	*Hardiness*
	Powerlessness	*Resilience*
	*Sense of coherence*	Emotional vitality
	Depression	*Shame*
	*Hopelessness*	*Guilt*
	Hostility	*Regret*
	Anger	*Emotion regulation*
	*Type A behavior*	*Emotional control*
	Anxiety	Well being
	*Environmental demands*	*Altruism*
	Life events	*Sadness*
	Stress	Fear
	Mood states	*Neuroticism*
	Positive affect	*Boredom*
	Negative affect	*Capacity to care*
	*Optimism*	Life satisfaction
	Self-esteem	Spirituality
	Quality of life	Caregiver burden
	Loneliness	Acculturation
	Social support	Discrimination
Intervention	Intervention	Reminiscence therapy
	Treatment	Assertiveness training
	Prevention	Strengths-based
	Exercise	Positive psychology
	Physical activity	Social support
	CBT	Spirituality
	Psychotherapy	Complementary and alternative medicine
	Life review	Integrated medicine stress management
	Meditation	Anger management
	Mindfulness	Coping skills
Community based	Community	Primary care
	Home	Community health center
	Neighborhood	
Older adults	Older adults	Middle-Aged
	Aged	Limits of 40 and older (to include 50 and older)
	Elderly	
Study design	Clinical trial	Experimental replication
	Multicenter study	Follow-up study
	Randomized controlled trial	Field study
	Randomized clinical trial	Non-clinical case study
	Evaluation studies	Qualitative study
	Clinical case study	Quantitative study
	Empirical study	

**Note*: We did not find any physical activity, social support, or skills training intervention studies that targeted the emotional health outcomes in italics*.

#### Expert panel and review methods

This review was guided by an eight-member expert panel of health services and mental health researchers from around the United States representing psychology, psychiatry, geriatrics, public health, and social work. The systematic review methods were derived from the *Guide to Community Preventive Services* (“The *Guide*”) (9, 10) and the systematic literature review of strategies to address late-life depression (11), using a formal process to identify relevant studies, assess their quality, and summarize the evidence. We searched the peer-reviewed literature through June 2008 and updated the search in June 2012 using PubMed (www.ncbi.nlm.nih.gov), CINAHL (http://www.ebscohost.com/academic/cinahl-plus-with-full-text/), and PsycINFO (www.apa.org/pubs/databases/psycinfo/index.aspx) databases. Subject headings and text words reflected our study aims, including key concepts of “emotional health,” “older adults,” “community based,” and “intervention”; specific terms are provided in Table 1. References to meta-analyses and review papers were also examined, and expert panelists reviewed the citations of included articles.

### Study selection

Study inclusion criteria were (1) published data on populations aged 50 years and older, (2) community-based sample and setting, (3) clearly described intervention; and (4) “emotional health” operationalized using the list of constructs determined by the expert panel (see Table 1). There were no restrictions on sample size or study design. Articles were excluded if they: were not available in English; reported only a review of the literature, meta-analysis, or commentary; focused exclusively on inpatient or institutionalized persons. We included articles from any country as long as they were published in English. We excluded interventions that targeted depression given the overlap with a previously conducted review focusing on late-life depression (11). The emotional component of quality of life measures was included [e.g., the role emotional subscale of the SF-36 (12)]; however, physical subscales were excluded. For studies aimed at addressing outcomes not strictly emotional in nature (e.g., spirituality, caregiver burden), we required the inclusion of at least one other emotional health outcome from the list of constructs.

We used a two-step screening process evaluating abstracts and where necessary full text to assess whether articles met inclusion criteria. A standardized form was used to systematically collect key data from each article, including study design, sample size, intervention setting, outcome measures, results, and indicators of study quality. Data were compiled in summary tables that the expert panel used for the evidence rating. As employed in our prior review (11), we grouped articles into intervention type-emotional health outcome pairings to categorically rate the evidence. For example, skills training interventions aimed at reducing anxiety were paired together.

Expert panel members rated the quality and effectiveness of each intervention–outcome pairing (Table 2). For quality rating, panel members independently rated the set of studies for each intervention–outcome pairing as *Good, Fair*, or *Limited*. Because few pairings received a vote of “good,” the good and fair categories were collapsed into a single category labeled “at least fair” quality. For effectiveness ratings, the panel members independently rated each intervention–outcome pairing as *Strong, Sufficient*, or *Insufficient*. For any pairing rated as insufficient, panel members were asked to record whether the rating was due to (1) an insufficient number of available studies or (2) a sufficient number of available studies but an insufficient amount of data to determine effectiveness. As established at the start of the review process, final determination of quality and effectiveness was based on 80% agreement among panel members. The panel met to discuss areas of disagreement and panel members were allowed to change their votes after the discussion; however, they were not required to reach consensus.

**Table 2:** **Indicators of quality and effectiveness for rating the evidence**.

Quality indicators	Effectiveness indicators
Well-described study population and intervention	Study quality
Sampling	Study design
Inclusion/exclusion criteria	Number of studies
Data analysis	Consistency across studies
Interpretation of results	Statistical results

## Results

**Figure 2:**
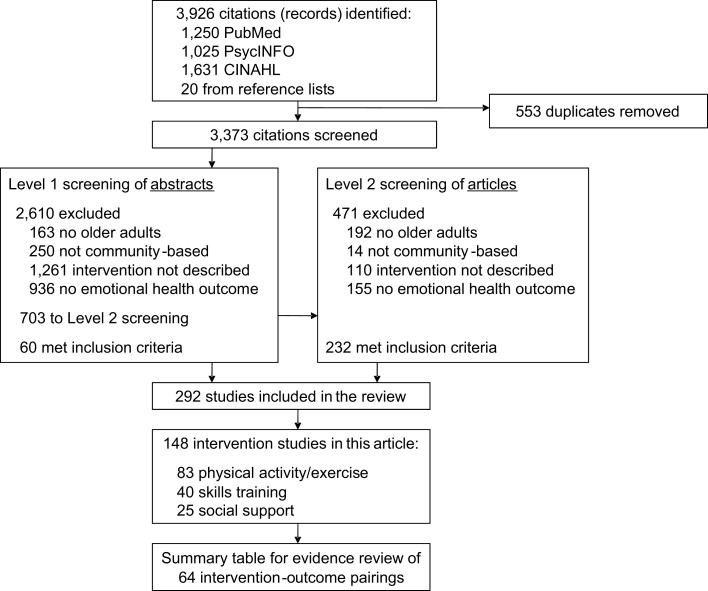
**Literature review flow chart**.

A total of 3,926 articles were identified in the initial search (1,250 from PubMed, 1,025 from PsycINFO, 1,631 from CINAHL, and 20 from reference lists of review articles or meta-analyses). 553 articles were duplicates and were eliminated (Figure 2). Two hundred ninety-two articles were eligible for inclusion, with the majority of the ineligible being excluded due to having too young of a sample size, not being an intervention study, or not having an emotional health outcome. Of the 292 eligible articles, the expert panel focused on three types of interventions relevant to public health practice and with ample studies for rating the evidence. These comprised a total of 148 of the 292 found articles: physical activity and/or exercise (*n* = 83), skills training (*n* = 40), and social support (*n* = 25) (Table 3). More than half of the studies (57%) were from the US or Canada, 19% were from European studies, 12% were from Australia or New Zealand, and 11% were from Asia. Thirty-nine percent of the articles specified that a theoretical framework that was used to inform the development of the intervention – one-third of the studies that evaluate an exercise or a social support intervention used a theoretical framework, while two-thirds of skills training interventions used a theoretical framework. Across interventions, the most common frameworks used across interventions were social cognitive theory, self-efficacy, and social learning theory. Other theories include the progressively lowered stress threshold model, the self-care deficit nursing theory of Orem, mindfulness meditation, self-management model of illness behavior, stress and coping theoretical framework, stress process models of caregiving, the transtheoretical model of behavior change, stages of change, negotiated adherence model, motivational interviewing, transforming hope theory, and Yalom group theory.

**Table 3:** **Intervention–outcome pairings for skills training, social support + skills training, and physical activity interventions**.

Intervention	Emotional health outcome	# Of studies (*n*)^a^	Quality rating	Effectiveness rating
Skills training	Anger	3 (258) (13–15)	At least fair	Insufficient (no consensus)
Skills training	Anxiety	11 (1,346) (13, 16–25)	At least fair	Sufficient
Skills training	Mood	5 (988) (13, 18, 26, 27, 76)	At least fair	Insufficient (no consensus)
Skills training	Other positive outcomes	2 (99) (29, 145)	At least fair	Insufficient (not enough studies)
Skills training	Psychological well being/distress	4 (1,449) (31, 32, 124, 142)	At least fair	Insufficient (multiple studies, inconclusive data)
Skills training	Quality of life	11 (1,417) (17, 22, 29, 31, 35–41)	At least fair	Sufficient
Skills training	Self-efficacy	16^b^ (3,735) (14, 15, 18, 20, 24, 26, 27, 30, 35, 39, 41–46, 175)	At least fair	Sufficient
Skills training	Spirituality	3^b^ (283) (23, 27, 65, 148)	Limited	Insufficient (not enough studies)
Skills training	Stress	4^b^ (500) (39, 45, 46, 98, 142)	At least fair	Insufficient (multiple studies, inconclusive data)
Social support	Anxiety	3^b^ (502) (34, 93, 135, 138)	At least fair	Insufficient (no consensus)
Social support	Loneliness	2 (313) (72, 108)	Limited	Insufficient (not enough studies)
Social support	Mood	2^b^ (144) (72, 109, 113)	Limited	Insufficient (not enough studies)
Social support	Other positive outcomes	1 (39) (83)	Limited	Insufficient (not enough studies)
Social support	Psychological well being/distress	5^b^ (704) (31, 34, 89, 128, 135, 139)	At least fair	Insufficient (multiple studies, inconclusive data)
Social support	Quality of life	3^b^ (450) (31, 34, 135, 138)	At least fair	Insufficient (no consensus)
Social support	Self-efficacy/locus of control	1 (39) (83)	Limited	Insufficient (not enough studies)
Social support + skills training	Anxiety	5 (580) (54, 63, 70, 100, 143)	At least fair	Insufficient (multiple studies, inconclusive data)
Social support + skills training	Mood	1 (144) (70)	At least fair	Insufficient (not enough studies)
Social support + skills training	Other negative outcomes	2 (415) (47, 82)	At least fair	Insufficient (not enough studies)
Social support + skills training	Other positive outcomes	3^c^ (58) (33, 66)	At least fair	Insufficient (no consensus)
Social support + skills training	Psychological well being/distress	6 (1,041) (14, 47, 70, 82, 144, 174)	Limited	Insufficient (multiple studies, inconclusive data)
Social support + skills training	Quality of life	3^b^^,^^c^ (393) (66, 109, 113, 121)	At least fair	Insufficient (no consensus)
Social support + skills training	Self-efficacy/locus of control	3 (408) (65, 70, 121)	At least fair	Insufficient (no consensus)
Motivation/counseling	Mood	1 (86) (103)	At least fair	Insufficient (not enough studies)
Motivation/counseling	Other positive outcomes	2 (969) (71, 79)	At least fair	Insufficient (No consensus)
Motivation/counseling	Quality of life	4 (850) (52, 64, 71, 120)	At least fair	Insufficient (Multiple studies, inconclusive data)
Motivation/counseling	Self-efficacy/mastery	5 (567) (71, 79, 92, 112, 176)	At least fair	Insufficient (multiple studies, inconclusive data)
Motivation/counseling	Stress	2 (1,712) (79, 118)	At least fair	Insufficient (no consensus)
Aerobic: walking	Anxiety	3 (507) (59, 102, 146)	At least fair	No Consensus (btw sufficient and insufficient, multiple studies)
Aerobic: other aerobic activities	Anxiety	4 (361) (57, 73, 114, 136)	At least fair	Insufficient (multiple studies, inconclusive data)
Aerobic: walking	Caregiver burden	1^b^ (100) (60, 102)	At least fair	Insufficient (not enough studies)
Aerobic: walking	Mood	2 (170) (107, 147)	At least fair	Insufficient (no consensus)
Aerobic: walking	Other positive outcomes	1 (582) (101)	At least fair	Insufficient (not enough studies)
Aerobic: other aerobic activities	Other positive outcomes	2 (150) (57, 114)	At least fair	Insufficient (not enough studies)
Aerobic: walking	Quality of life	6 (1,273) (56, 101, 104, 123, 130, 147)	At least fair	Insufficient (multiple studies, inconclusive data)
Aerobic: other aerobic activities	Quality of life	6 (823) (51, 57, 117, 134, 151, 179)	At least fair	Insufficient (multiple studies, inconclusive data)
Aerobic: walking	Psychological distress and well-being	91 (28)	At least fair	Insufficient (not enough studies)
Aerobic: other aerobic activities	Psychological distress and well being	101 (136)	At last fair	Insufficient (not enough studies)
Aerobic: walking	Self-efficacy/mastery/locus of control	1 (32) (62)	NC	Insufficient (not enough studies)
Aerobic: Other aerobic activities	Self-efficacy/mastery/locus of control	3 (231) (56, 106, 114)	NC	Insufficient (no consensus)
Aerobic: walking	Stress	2^b^ (457) (59, 60, 102)	At least fair	No consensus (btw sufficient and insufficient, not enough studies)
Strength/resistance	Anxiety	1 (42) (129)	At least fair	Insufficient (not enough studies)
Strength/resistance	Fear of falling	2 (94) (48, 150)	At least fair	Insufficient (no consensus)
Strength/resistance	Loneliness	1^b^ (32) (84, 129)	At least fair	Insufficient (not enough studies)
Strength/resistance	Mood	2 (144) (69, 153)	At least fair	Insufficient (no consensus)
Strength/resistance	Psychological well being/distress	2 (124) (134, 153)	At least fair	Insufficient (not enough studies)
Strength/resistance	Quality of life	13^b^ (1,000) (28, 68, 75, 84, 115, 119, 122, 126, 132–134, 137, 153, 177)	At least fair	Insufficient (multiple studies, inconclusive data)
Strength/resistance	Self-efficacy/locus of control	7^b^ (442) (75, 115, 126, 129, 132, 137, 153, 177)	At least fair	Insufficient (multiple studies, inconclusive data)
Stretch/flexibility/balance/agility	Anxiety	1 (88) (96)	NC	Insufficient (not enough studies)
Stretch/flexibility/balance/agility	Fear of falling	2^b^ (422) (53, 90, 181)	At least fair	No consensus (btw sufficient and insufficient)
Stretch/flexibility/balance/agility	Mood	5 (307) (49, 87, 95, 116, 147)	At least fair	Insufficient (no consensus)
Stretch/flexibility/balance/agility	Other positive outcomes	1^b^ (200) (53, 182)	At least fair	Insufficient (not enough studies)
Stretch/flexibility/balance/agility	Psychological well being/distress	1^b^ (200) (53, 182)	At least fair	Insufficient (not enough studies)
Stretch/flexibility/balance/agility	Quality of life	8^b^ (853) (48, 51, 53, 87, 94, 96, 132, 147, 181)	At least fair	Insufficient (multiple studies, inconclusive data)
Stretch/flexibility/balance/agility	Self-efficacy/mastery/locus of control	5 (465) (48, 90, 94, 95, 132)	At least fair	No consensus (btw strong, sufficient, insufficient)
Stretch/flexibility/balance/agility	Stress	1 (39) (95)	NC	Insufficient (not enough studies)
Combination	Anxiety	3 (485) (91, 180, 182)	At least fair	Insufficient (no consensus)
Combination	Fear of falling	2 (200) (85, 88)	At least fair	Insufficient (no consensus)
Combination	Mood	3 (257) (81, 97, 173)	At least fair	Insufficient (no consensus)
Combination	Other positive outcomes	3 (459) (91, 131, 178)	At least fair	Insufficient (multiple studies, inconclusive data)
Combination	Psychological well being/distress	6 (748) (97, 131, 133, 180, 182, 184)	At least fair	Insufficient (multiple studies, inconclusive data)
Combination	Quality of life	16 (7,492) (55, 61, 78, 80, 81, 85, 86, 88, 97, 99, 110, 111, 149, 152, 182, 183)	At least fair	Insufficient (multiple studies, inconclusive data)
Combination	Self-efficacy/mastery/locus of control	5^b^ (654) (77, 92, 105, 125, 127, 183)	At least fair	Insufficient (multiple studies, inconclusive data)
Combination	Stress	1 (187) (180)	NC	Insufficient (not enough studies)

*NC, no consensus*.

*^a^Article citations for each intervention–outcome pairing are provided in this column. Some of the 148 studies are listed in more than one intervention–outcome pairing*.

*^b^Several studies are reported in more than one article (e.g., article #40 and article #41 describe the same study using different analyses)*.

*^c^Article #62 reported on two different positive outcomes, self-esteem and life satisfaction*.

The physical activity and/or exercise interventions included aerobic activity, strength training, balance and flexibility interventions, motivational strategies, and a combination of exercise types. The skills training group included self-management [e.g., Chronic Disease Self-Management Program (CDSMP)], psycho-education, anger management, and stress management interventions. The social support group included interventions targeting direct or indirect provision of social support (e.g., interventions designed to improve ability to obtain support).

The 148 studies were subsequently grouped into 64 intervention type–outcome pairings, or categories, for rating the evidence, such as social support interventions aimed at elevating mood (Table 3). For quality, 53 (83%) of the intervention–outcome pairings were rated as having “at least fair” quality; only 11% of these had good quality. For effectiveness, a majority of pairings (89%) were deemed to have insufficient evidence, due to lack of studies (two or fewer) or inconclusive evidence (mixed results within or across studies). Herein, we will report findings for the three intervention–outcome pairings for which sufficient evidence was found. For further information about categories not presented or on detailed summary data tables, please contact the corresponding author.

### Intervention–outcome pairings with sufficient evidence

#### Skills training

Sufficient evidence was found for effectiveness of skills training interventions to reduce anxiety and to promote quality of life and self-efficacy (from a total of 38 studies). These studies were rated as having “at least fair” quality. Of these studies, 11 were aimed at reducing anxiety, of which four involved randomized controlled trials (RCT). They involved 1,346 participants and represented a diverse subject population (e.g., caregivers and people with breast cancer, heart disease, or arthritis). Only three studies reported dropout rates, and in two of these, that rate was below 20%. Study duration varied from 2 to 12 months, although generally the active phase ranged from 6 to 8 weeks.

The report by López et al. (16) focused on caregivers in which the majority of care was provided to persons living with dementia (80%).They found a 38% decrease in mean anxiety score in the Hospital Anxiety and Depression Scale (HADS) (154) for traditional format skills training (60 min weekly over a period of 8 weeks) involving cognitive behavioral approaches, assertiveness training, self-esteem building exercises, and problem-solving skills training. The other studies using the HADS found a 10–20% decrease in anxiety scores after intervention (17, 18). The Williams (19) study of 71 women with breast cancer found no effect for a 20-min audiotape to teach skills for decreasing sleep, anxiety, and fatigue problems encountered during chemotherapy. Two non-randomized, controlled trials did not show a significant effect. One focused on asthma self-management and another focused on Chinese older adults with history of depression or anxiety, although there was a non-significant trend toward effectiveness (*p* < 0.10) (20, 21). Five single-group studies revealed mixed results (13, 22–25).

Eleven additional skills training studies aimed at emotional health as measured by the subscales of a quality of life measure such as the SF-36. There were eight RCTs, two quasi-experimental studies, and one single-group study. A total of 1,417 participants were included in these studies, with sample sizes ranging from 35 to 320, averaging between 75 and 100 participants. The duration of the interventions ranged from 1 week to 8 months, averaging between 6 and 8 weeks. Interventions included both group and individual-level activities. Dropout rates of less than 20% were reported for all but two studies. Seven studies [five RCTs (17, 35–39) and one non-RCT (40)] reported statistically significant improvements in at least one emotional health subscale of the SF-36 Quality of Life measure. Specifically, statistically significant improvements were reported for the *vitality and role limitations emotional* SF-36 subscales for Barnason et al.’s (35) phone-based home communication intervention for older adults with ischemic heart failure (*p* < 0.01). Similarly, Grant et al.’s (36) social problem-solving phone partnership for adult caregivers of stroke survivors improved quality of life subdomains (*p* = 0.013). McHugh et al.’s (17) share care health education and motivational interviewing program for adults waiting for elective CABG (*p* = 0.000), and Wallace et al.’s (37) nurse visit to develop a customized health plan for older adults exercising at a local senior center were found to be effective (*p* = 0.02). No significant improvements in *vitality* were found for Markle-Reid et al.’s (26, 38) individual-level program to bolster personal and environmental resources of frail, older home care clients although this study did find improvement using the *role limitation emotional* subscale. In addition to Grant et al. (36), Markle-Reid et al. (38), McHugh et al. (17), and Wallace et al. (37) studies, Hughes et al. (39) study of a workshop intervention for women with self-reported disabilities all reported significant improvements in the SF-36 *mental health* subscale. Furthermore, two studies (38, 40) found significant improvements in the *mental health composite* SF-36 measure (including vitality, mental health, and role limitation emotional). Significant improvements were demonstrated in two studies using emotional health subscales of quality of life-specific measures for older adults with heart failure (13, 22–25, 31, 35–38). The remaining two studies (29, 41) did not find improvements in emotional health subscales of different quality of life measures.

Sixteen skills training intervention studies were directed at improving self-efficacy. These studies included 11 RCTs, two observational studies, and three single-group studies. Seven of the studies were of interventions using the CDSMP. A total of 3,735 participants received skills training interventions, with sample sizes ranging from 33 to 728. Study duration averaged 6 to 8 weeks. Dropout rates, reported in half the studies, were less than 20%. The frequency of the skills training interventions was rarely reported. When reported, adherence to the intervention was typically less than 80%. The interventions were delivered most often in a group format and the control groups were generally usual care and wait-list control conditions. Eight of the 11 RCTs (14, 15, 26, 27, 35, 42–46) reported significant improvements in self-efficacy; three of the significant studies used CDSMP (15, 42, 45). Four of the five non-RCT studies (15, 20, 24, 32) also demonstrated significant improvements in self-efficacy. All but Smith et al. (20) study were single-group designs with 20–32% dropout rates.

#### Exercise and social support

The expert panel did not find sufficient evidence for either exercise or social support interventions to improve emotional health.

### Other intervention–outcome pairings

#### Skills training

The expert panel found insufficient evidence for 20 other skills training interventions that focused on other emotional health outcomes such as mood and stress. Most of these pairings were of at least fair quality. In addition, 82 studies were found that reported on the effects of physical activity and/or exercise on emotional health outcomes, and 25 studies looked at social support interventions. There was insufficient evidence of effectiveness for most of these intervention–outcome pairings and the panel rated most of the pairings as at least fair quality.

#### Exercise and physical activity

The expert panel did not reach consensus for several physical activity and exercise intervention–outcome pairings. First, the panel was split between ratings of sufficient and insufficient for stretching, flexibility, balance, or agility interventions to decrease fear of falling. Second, panel members did not agree on whether there was sufficient evidence that stretching, flexibility, balance or agility interventions improved self-efficacy, mastery, or locus of control. Panel members raised concerns about limited numbers of studies for any single outcome and about mixed results observed across the study outcomes. Finally, the expert panelists were split between evidence ratings of sufficient and insufficient for walking interventions that targeted anxiety or stress. Insufficient evidence was found for all other exercise and physical activity interventions.

#### Social support

The expert panel found insufficient evidence that the reviewed social support interventions improved emotional health.

## Discussion

This review examined three broad types of interventions designed to promote emotional health: physical activity and/or exercise, skills training, and social support. Among the interventions rated as having at least fair quality and sufficient evidence, we found that skills training interventions reduced anxiety; enhanced self-efficacy; and improved vitality, role functioning related to emotional limitations, and emotional health as measured in quality of life subscales. Skills training interventions are theorized to promote positive domains of emotional health through cognitive reframing, strengthening coping resources, and increasing the amount of support (or quality of support). We acknowledge that skills training may improve emotional health through improved self-efficacy, though the panel chose to view self-efficacy as its own emotional health domain. These interventions are designed for older adults with chronic conditions (e.g., arthritis, heart disease, physical disabilities) or informal caregivers (e.g., spouses, adult children) of older adults coping with dementia, stroke survivors, or mental illness making them quite generalizable. These populations were targeted by these interventions because chronic conditions or caregiving responsibilities increase the need for skills training, support, information, and resources.

The CDSMP was used as an intervention in seven of the skills training studies that showed sufficient evidence for improving quality of life or self-efficacy or decreasing anxiety. CDSMP has been shown to enhance stress management techniques, improve communication with physicians, increase confidence in ability to manage the condition, and improve role function (32, 42, 155–159). Improving self-management skills has been shown to impact other aspects of participants’ lives, such as their ability to manage their emotions, choose healthy foods and exercise activities, and activate their social network (158). This review is limited by its end date of June 2008. While it is beyond the scope of this project to conduct an updated systematic literature review, we recently searched for other review papers on skills training, exercise and/or physical activity, and social support interventions to promote emotional health. We found two review papers (160, 161) that reported similar findings as we report above, namely, sufficient evidence for skills training interventions impact on self-efficacy and quality of life and insufficient evidence for other emotional health outcomes. We also searched for intervention studies for those areas where sufficient evidence was found. Our search yielded 10 recently published articles (162–171), none of which reported different findings than reported above.

We defined “insufficient evidence of effectiveness” in two ways: either there were not enough studies of at least fair quality, or there were multiple studies with inconclusive data. Insufficient evidence did not mean that interventions were clearly ineffective. Very few intervention–outcome pairings were rated as at least fair quality. The expert panel identified the following common quality limitations: lack of descriptive information about the interventions, limited information about the statistical methods and analyses, and small sample sizes or underpowered studies. Additionally, features of some of the study designs made it difficult to detect changes in emotional health. For example, many studies included emotional health outcome measures that may not be responsive to small changes from programs of limited intensity and duration, and sampling “emotionally healthy” subjects that created ceiling effects. In fact, many of the reviewed aerobic physical activity interventions did not meet current national guidelines (140) for 150 min per week of moderate-intensity activity (though all reviewed strength/resistance interventions did meet existing criteria of 2 days per week).

Our review included a wide range of emotional health constructs. Some outcomes were entirely emotional (e.g., anxiety), whereas others included a mix of cognitive, emotional, and behavioral domains (e.g., self-efficacy). In addition, some studies included emotional health outcomes as their primary outcomes, whereas others included emotional health as intermediate outcomes or mediators of other health outcomes. Finally, there was a dearth of intervention studies on certain emotional health constructs, such as hopelessness, shame, guilt, regret, fear, neuroticism, boredom, positive energy, contentment, hardiness, resilience, emotional stability, emotional regulation/control, altruism, capacity to care, and happiness. In particular, positive constructs were underrepresented in the available literature. We were not surprised that there was limited evidence on interventions to promote emotional health, and particularly any studies lacking in positive emotional health constructs given the tendency (up until recently) to focus on disease prevention over health promotion. We anticipate that more research will include emotional health outcomes as models such as the socio-ecological model (67, 172) and guidelines such as the Public Health Action Plan to Integrate Mental Health Promotion and Mental Illness Prevention with Chronic Disease Prevention, 2011–2015 (74) emphasize the importance of emotional health in the larger public health goals.

Future research needs to address these quality concerns by attending to limitations with both internal and external validity. One way to do so is to use the RE-AIM framework, a conceptual approach for evaluating the translation of research into practice in “real-world” settings (141). RE-AIM stands for reach, effectiveness, adoption, implementation fidelity, and maintenance – five areas, which, if addressed, ensure that essential program goals are retained during the implementation process, resulting in greater external validity. More research is also needed to investigate the longer term, maintenance effects of interventions to promote positive emotional health, and address illness-related domains in older adults as most of the studies here were of short-term effectiveness. The prominence of theories such as social cognitive theory, social learning theory, and self-efficacy theory in those interventions with sufficient evidence may also be helpful to consider in future intervention design and development as they may have contributed to the optimization of participants’ quality of life and self-efficacy and minimization of anxiety symptoms.

Despite the gaps in the current research, our systematic review provides important information about interventions that can promote emotional health outcomes in community-dwelling older adults. Specifically, we found that skills training interventions resulted in improvements in both illness-related (anxiety) and positive (quality of life and self-efficacy) domains of emotional health. Given that more than one in four Americans lives with two or more concurrent chronic conditions, the challenges of managing multiple chronic conditions among the growing numbers of older persons are significant (50). One of the overarching goals of the U.S. Department of Health and Human Services’ Strategic Framework (58), *Optimum Health and Quality of Life for Individuals with Multiple Chronic Conditions*, is to *“*maximize the use of proven self-care management and other services by individuals with multiple chronic conditions.” As shown in this review, skills training interventions can offer important benefits in the realm of promoting emotional health in older adults. Given the expanding proportion of older adults in the US and globally, we hope this review will help in addressing some of challenges identified in this important area of study.

## Conflict of Interest Statement

The authors declare that the research was conducted in the absence of any commercial or financial relationships that could be construed as a potential conflict of interest.

This paper is included in the Research Topic, “Evidence-Based Programming for Older Adults.” This Research Topic received partial funding from multiple government and private organizations/agencies; however, the views, findings, and conclusions in these articles are those of the authors and do not necessarily represent the official position of these organizations/agencies. All papers published in the Research Topic received peer review from members of the Frontiers in Public Health (Public Health Education and Promotion section) panel of Review Editors. Because this Research Topic represents work closely associated with a nationwide evidence-based movement in the US, many of the authors and/or Review Editors may have worked together previously in some fashion. Review Editors were purposively selected based on their expertise with evaluation and/or evidence-based programming for older adults. Review Editors were independent of named authors on any given article published in this volume.
